# Research progress analysis of live streaming commerce based on CiteSpace

**DOI:** 10.1016/j.heliyon.2024.e36029

**Published:** 2024-08-09

**Authors:** Yi Huang, Nurkhalida Makmor, Siti Hajar Mohamad

**Affiliations:** a Graduate School of Management, Management and Science University, Shah Alam, 40100, Malaysia; b School of Artificial Intelligence and Big Data, Henan University of Technology, Zhengzhou, 450001, China; c Faculty of Business Management and Professional Studies, Management and Science University, Shah Alam, 40100, Malaysia

**Keywords:** Live streaming commerce, Bibliometric analysis, CiteSpace, Research progress

## Abstract

In recent years, live streaming commerce (LSC) has become a popular research topic. However, current studies on LSC are relatively insufficient, and analyses have generally focused on its specific aspects, lacking a comprehensive and systematic perspective. Hence, this study utilises CiteSpace to undertake a visual bibliometric analysis aimed at delineating the knowledge framework and evolution of LSC and indicates future research directions to provide a comprehensive picture of the development of this dynamic field over the past six years. The results show that LSC is a thriving subject with several growing annual publications. Additionally, a strong collaboration exists between institutions and authors. Further, ‘influence’, ‘consumer behaviour’ and ‘consumer purchase intention’ are more popular in this domain and have assumed a leading position in recent research. Moreover, novel research trajectories have emerged, indicating interdisciplinary integration within the field. This study is innovative as it combines live streaming with commerce, analyses six years of literature, and builds an accurate and comprehensive knowledge framework within this domain. By identifying current gaps, this study contributes to the literature by addressing prior study limitations, enriching the knowledge base, providing crucial research directions for future exploration, and inspiring scholars to efficiently find research topics.

## 1 Introduction

As economic globalisation progresses, the interconnection of economies between nations intensifies, ushering in new prospects for global economic advancement [1]. The rapid evolution of information and communications technology has propelled live streaming commerce (LSC) into an era of dynamic growth on a global scale [2,3]. The digital economy is a new economic model in human history [4], and LSC is a new marketing model in the digital economy era. Represented by Amazon Live and Taobao Live, the LSC model has become an essential part of e-commerce [5]. Previously, products were only displayed through texts and pictures and later developed into live streamers through live streaming to show and interact with consumers in real-time [6]. The shopping experiences of LSC users are usually highly interactive, entertaining, authentic, and visible [7]. Notably, LSC refers to a business form wherein merchants use e-commerce platforms to display products to consumers in a live stream to enable them to better understand and complete the product’s purchase [8]. Users can purchase products they see in a live stream immediately [9], facilitating the buying process. However, the dullness of live commercial content [10], its apparent sales orientation [11], and lengthy product demonstrations in LSC are becoming increasingly prominent [12]. This leads to poor user experience, affecting consumers’ buying decision behaviours [13]. Considering the digital economy’s rapid growth, addressing LSC sustainability is crucial [14].

The number of primary studies on LSC has significantly grown in recent years, covering various topics from different perspectives. Previous studies have summarised the following points: (1) Consumer behaviour in the LSC context. Scholars have explored consumers’ purchase intention [15,16], impulse buying behaviour [17,18], continuous buying behaviour [19], panic buying behaviour [20], and engagement behaviour [[21], [22], [23]]. Some scholars have summarised the literature on LSC purchasing behaviour from the perspectives of live streamers [[24], [25], [26], [27]], platforms [28,29], merchants [30], and consumers [31]. (2) User experience technologies. Previous scholars have summarised how to use artificial intelligence, big data, and other technology applications to improve the consumer experience [[32], [33], [34]]. (3) Product sales strategy. Researchers have systematically reviewed product pricing [35], product display [36], and quality strategies [37]. Moreover, some studies have explored LSC comprehensively, suggesting that future research be conducted in four areas—namely, novelty, context, methodology, and LSC theory [38]. Past studies have provided numerous insights into LSC, inspiring us to explore further.

Nevertheless, these studies have some limitations. Current studies focus on discrete aspects and, therefore, overlooked other critical aspects, such as the full range of research developments and trends in this domain, which could have contributed more to a comprehensive understanding and expansion of LSC knowledge. For instance, studies have focused on consumer purchasing behaviour. (1) A comprehensive perspective is required to understand the general topic that LSC encompasses as a comprehensive overview and examination of developmental trends in this research field is lacking [39]. (2) Past studies have predominantly focused on LSC for a limited period; a dynamic framework is necessary to understand how this topic has drastically evolved over the past six years. (3) Visual results are needed to describe the evolution and trends in this field to enable readers to easily understand the topic.

Therefore, summarising the research results on LSC in the field from a lasting dynamic perspective is necessary. It is essential to comprehend the development of the structure and identify the current focal points, progress, and emerging domains in LSC to examine these aspects effectively. Moreover, current publications seem to overlook these inquiries, much less visualise the results. Thus, it is necessary to summarise the research results and examine the visualised results’ evident trends to understand the current hotspots, advance fresh insights and perspectives, and provide substantial references for researchers and practitioners.

Hence, to provide readers with a more comprehensive and immediate understanding of the field’s evolution and simplify the understanding of research frontiers and hotspots, we conducted a visual bibliometric analysis of the LSC literature over the past six years (01/2018–01/2024). The following research questions are addressed.

Q1 How many articles are published every year, and what is the current status of popular institutions and core authors?

Q2 What are the research hot-spots and evolution paths of LSC?

Q3 What implications does existing research have for future LSC research?

This study’s novelty is as follows: (1) In terms of knowledge, we explored the research details of the number of publications, collaborations, and co-occurrence in the field over the past six years to comprehensively and accurately elucidate the current status, progress, evolution, and trends in this domain. (2) Theoretically, we present the results using visual figures that explain the content covered by LSC simply and directly, thus demonstrating the research results and constructing a new knowledge framework to help readers more clearly understand the logical structure of LSC research. (3) In practice, we clarify the characteristics of future research, enabling future researchers to quickly grasp specific basic knowledge and providing directions for scholars and practitioners.

In this paper, section 2 describes the data sources, data screening, and the tools employed. Section 3 summarises the results of the analysis of publication statistics and collaboration and co-occurrence networks. Section 4 provides this study’s implications and underscores specific features for future research. Section 5 provides the conclusions to help researchers understand the study in generic terms.

## 2 Materials and methods

### 2.1 Data sources and selection process

We sourced the data from the Social Sciences Citation Index (SSCI) and Science Citation Index Expanded (SCIE) in the Web of Science (WoS) Core Collection database (https://www.webofscience.com/wos/woscc/basic-search, accessed on 18 January 2024). This database was selected because of its importance as an essential information resource for the global scholarly community [40]. Furthermore, WoS database provides more comprehensive data and spans a longer timeframe, rendering it ideal for scientific research. Moreover, SCIE covers more than 9000 leading academic journals in the natural sciences, while SSCI covers more than 3000 influential journals in the social sciences. It contains high-quality articles recognised by peers [41], ensuring high-quality literature and making the results more convincing.

### 2.2 Search strategy

When collecting the literature, we excluded irrelevant studies, and set specific criteria to filter out less relevant ones. We used a topic search (TS) for highly relevant papers. The TS formula, which searches titles, abstracts, and keywords, is the most used search scope [42]. We conducted literature searches in the WoS database over a one-day period, specifically, up to 18 January 2024. To minimise bias owing to database updates, we completed searches within the same day. Considering the late rise of LSC and number of related studies, we used the TS search formula to avoid missing all literature on LSC in the WoS Core Collection database. The search strategy we initially used was TS = (live* commerce) OR TS = (ecommerce live broadcast*) OR TS = (e-commerce live stream*) OR TS = (live broadcast ecommerce) OR TS = (livestream* ecommerce) OR TS = (live* shopping) OR TS = (livestream* commerce). The search period was from 1 January 2018 to 18 January 2024. We chose the search period from 1 January 2018 to 18 January 2024 for several compelling reasons related to the emergence, growth, and maturation of LSC as a field of study: (1) LSC emerged as a commercial phenomenon in 2016 [23]. It started gaining traction as a viable business model, integrating live streaming technology with e-commerce. However, by 2018, the first academic articles began to appear, marking the formal academic recognition and investigation of this phenomenon. (2) Since 2018, LSC has achieved technological advancement, innovative business models, and diverse applications. By analysing this period, we can understand how early concepts have evolved and adapted to market needs and technological advances. Overall, we obtained 1606 documents.

### 2.3 Inclusion and exclusion criteria

For search accuracy and precise analysis results, we conducted the following data-cleaning steps.

(1). We excluded conference proceedings, early access papers, and letters, only retaining articles and review articles as they typically undergo rigorous peer review, which ensure their quality and credibility. Additionally, they provide comprehensive research data, methods, results, and discussions.
(2). We only chose English as the language of publication as it is widely used in academia, even among non-English speakers. Additionally, if the documents are in the same language, it is easier to analyse and highlight a large amount of key information.
(3). We conducted a thorough manual screening of all documents to further confirm the eligibility of the selected studies. Two domain researchers reviewed the included articles’ titles, abstracts and content to ensure that the inclusion criteria were fulfilled; they excluded unrelated articles. In case of disagreement between the reviewers, the senior author determined whether consensus was reached.

**Fig. 1:**
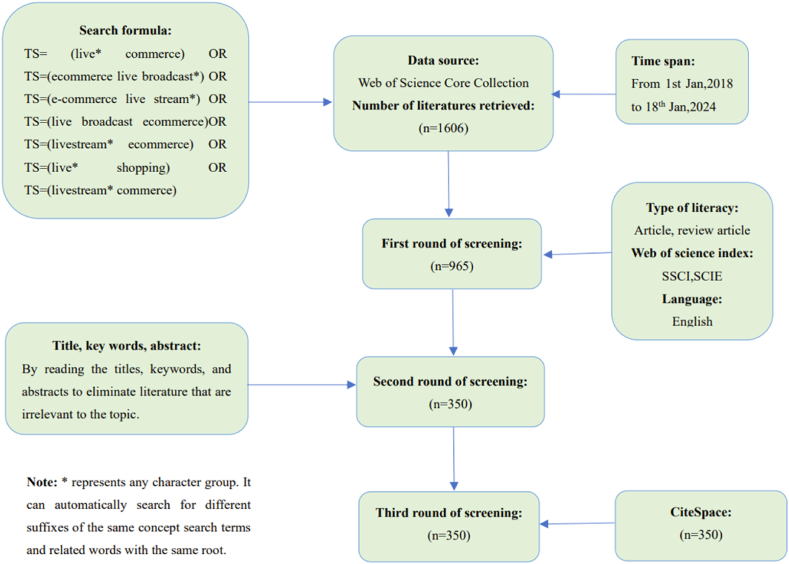
Flowchart of the data selection process.

In the first round of screening documents, we removed ‘Abstract Papers’, ‘Conference Proceedings Papers’, ‘Editorial Materials’, and ‘Letters’, and only retained ‘Articles’ and ‘Review Articles’. We selected SSCI and SCIE from the WoS Core Collection database. The language was English, and 965 articles were screened. In the second round of screening, after reading the titles, abstracts, and keywords, we excluded papers not related to this study’s subject disciplines, such as medicine and chemistry. Overall, we selected 350 articles for this study. Finally, we entered 350 articles into the bibliometric software for deduplication analysis and obtained 350 valid articles, which were designated for subsequent bibliometric review and in-depth analysis. Fig. 1 presents details regarding screening and eligibility.

### 2.4 Research methodology

Bibliometric analysis is an approach for measuring, tracking, and analysing academic literature using a set of quantitative methods [43]. By presenting bibliometric knowledge maps, researchers can gain a more intuitive and in-depth understanding of the current state of the field and scientifically capture trends [44]. As an exemplary bibliometric tool, CiteSpace excels in conducting multivariate, temporal, and dynamic citation visualisation analyses [45]. CiteSpace—developed by Prof. Chaomei Chen [46] possesses the following advantages: First, the clustering data analysis can identify focal areas of research, emerging frontiers, and trends in associated fields, providing insights for future research. Second, it is easy to download and data processing is time-saving and efficient [47].

**Fig. 2:**
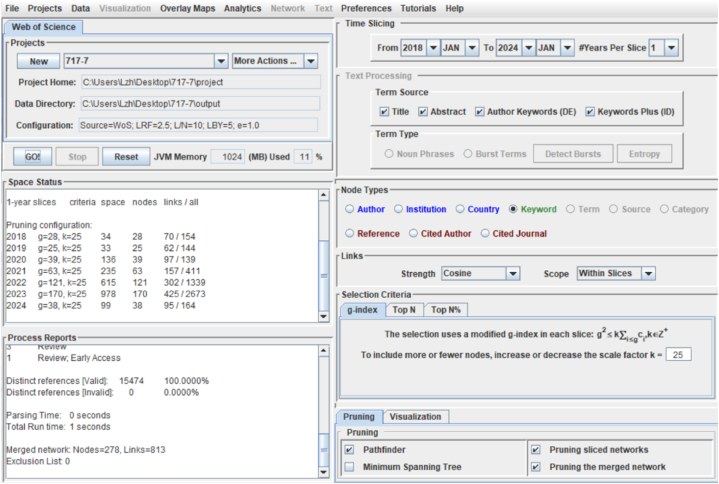
CiteSpace operation homepage.

Traditional literature review methods describe and summarise the topics, number of publications, and research areas in the literature [48]. Owing to the large amount of literature in this study’s original sample, the traditional literature review method is not applicable. Scientific bibliometric analysis is a literature review method that uses mathematical and statistical tools [49]. By presenting literature knowledge metric mapping, researchers can more intuitively understand the characteristics of the field and use the scientific method to track research dynamics in the field [50]. There are many bibliometric visualisation software tools, such as CiteSpace, VOSviewer, Histcite, and BioBERT. Among them, CiteSpace is user-friendly and one of the most used ones, but it is rarely used in the field of LSC. CiteSpace software can create knowledge graphs in specific research fields [51]. As an excellent bibliometric software, CiteSpace is suitable for multivariate, temporal, and dynamic literature visualisation and analysis [52]. This software can identify research hotspots, frontiers, and trends in related fields [53]. Thus, this study used CiteSpace version 6. 2. R4 to explore LSC research hotspots and trends. We conducted the following steps to obtain the results and visualise the network using CiteSpace: (1) Imported and deduplicated a file containing 350 articles exported from WoS database to finalise data from January 2018 to January 2024. (2) Selected the period from January 2018 to January 2024 with ‘one year per slice’ enabled. Set the text processing term source to title, abstract, author keywords (DE), and keywords plus (ID), with selection criteria set to Top 50. We used pruning options (pathfinder, prune sliced network, and prune the merge network) to enhance processing efficiency and graphic readability. (3) Selected author and institution node types to create a collaborative visualisation network, illustrating the collaborative relationships and identifying the most influential stakeholders in LSC research over the past six years. (4) Selected keyword node type to create a keyword co-occurrence network, helping to understand the evolution of research, current hotspots, and potential turning points. Fig. 2 illustrates the CiteSpace operation homepage.

Our research objectives are: (1) To illustrate the statistical features of the research field, including the number of annual publications on the topic of LSC, which helps us understand whether the field is in demand. (2) To examine the collaboration network involving institutions and authors to aid in comprehending the collaborative dynamics within the field. (3) To conduct co-occurrence analysis and cluster processing of keywords to evaluate the progression of LSC research and anticipate potential hotspots for future research. (4) To construct a knowledge framework based on previous research and propose future key research directions to stimulate scholars’ research interests.

## 3 Results

### 3.1 Publication statistical analysis

**Fig. 3:**
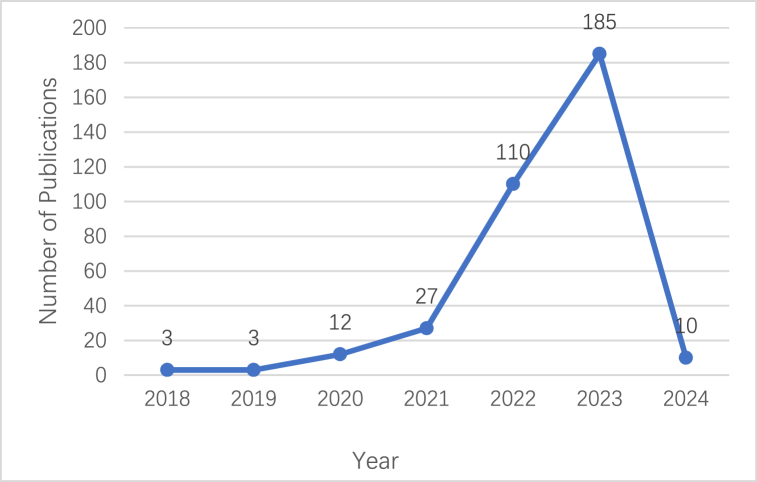
Number of annual publications from 2018 to 2024.

Changes in literature publications reflect the development in the field and future research trends [54]. Overall, 350 related studies published between 2018 and 2024 show fluctuating development trends over the past six years (Fig. 3), suggesting that academic interest and focus vary across the different stages of the process. The years 2018 and 2019 signified the nascent stages of the research field. The number of published articles was limited, totalling no more than four each year. This might be associated with the relatively novel emergence of LSC during this period and the potential limitations in research methodologies [55]. The number of articles issued increased from 2020 to 2023, a period of fluctuating growth. As LSC evolved and research methods became more diversified, the number of articles began to fluctuate and increase in 2020 [56]. This period signifies a phase of variable development in the study [57], reaching its pinnacle in 2023 with a peak of 185 articles published since 2018. This surge suggests widespread attention to the topic during this stage, indicating interest in related research [58].

In particular, the number of papers published in 2024 is comparatively lower than that of the preceding year. This is because we conducted the data collection in January 2024, and collected only part of the literature for 2024. However, we are optimistic that the number of publications will exceed that of the previous year. These results indicate the prevailing significance of LSC as a trending topic, we advocate that researchers should allocate more resources to this field. In conclusion, LSC has gained considerable attention over the past six years, and contemporary scholars need to continue expanding their research in this field.

### 3.2 Collaboration analysis

#### 3.2.1 Institution collaboration analysis

**Fig. 4:**
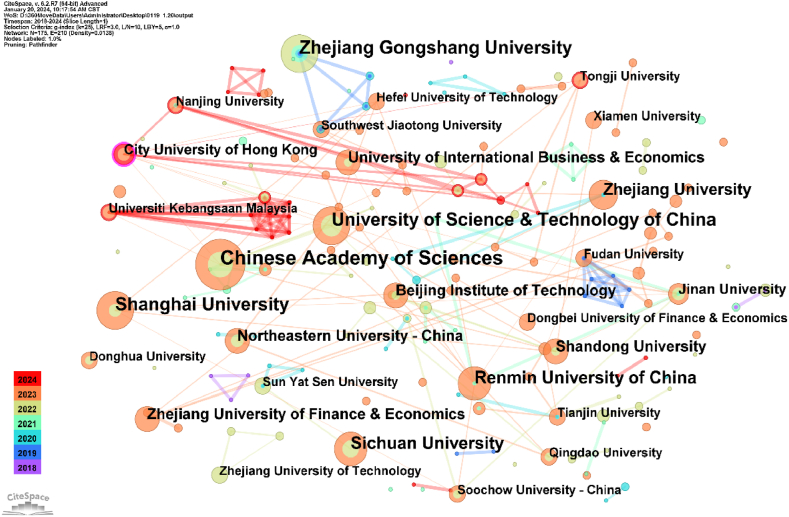
Visualisation of institution collaboration network.

The network map of the research institution cooperation elucidates the spatial distribution of research influences in this domain [59]. To identify the institutions driving the research, this study utilises collaboration network analysis integrated into CiteSpace software to extract network relationships among institutions [60]. This network visually illustrates the collaborative ties between institutions and serves as a scientific foundation for evaluating their scholarly contributions, offering valuable insights within the academic community [61]. We selected ‘Institution’ in the node-type panel and initiated the process to generate the research institution distribution network diagram depicted in Fig. 4 [62]. The node size reflects the number of journal articles published by the corresponding research institution [63]. The connections between nodes denote the strength of the collaboration, and the colours of the connecting lines represent the collaborative relationship during various periods [64].

As Fig. 4 depicts, the study sample comprised 175 nodes and 210 connections with a network density of 0.0138. The major institutions in the figure exhibit a relatively cohesive collaboration network. To analyse the results and cooperative relationships of institutions, we further data mined Fig. 4 to obtain the top 10 research institutions with the highest number of publications [65], as Table 1 indicates. The Chinese Academy of Sciences, University of Science and Technology of China, and Shanghai University published the most articles. Judging from the degree of collaboration between units, the Chinese Academy of Sciences and Renmin University of China had higher collaboration densities and more connections among institutions.

**Table 1:** Top 10 institution collaboration network.

Ranking	Institutions	Year	Count	Centrality
1	Chinese Academy of Sciences	2022	12	0.09
2	University of Science & Technology of China	2022	9	0.04
3	Shanghai University	2022	9	0.01
4	Zhejiang Gongshang University	2019	9	0.00
5	Renmin University of China	2021	8	0.09
6	Sichuan University	2022	8	0.00
7	Zhejiang University	2020	7	0.04
8	Shandong University	2022	6	0.06
9	Zhejiang University of Finance & Economics	2023	6	0.03
10	Northeastern University – China	2022	6	0.00

To summarise, different institutions have different research priorities. We recommend that scholars seek potential partner organisations based on their research questions.

#### 3.2.2 Author collaboration analysis

**Fig. 5:**
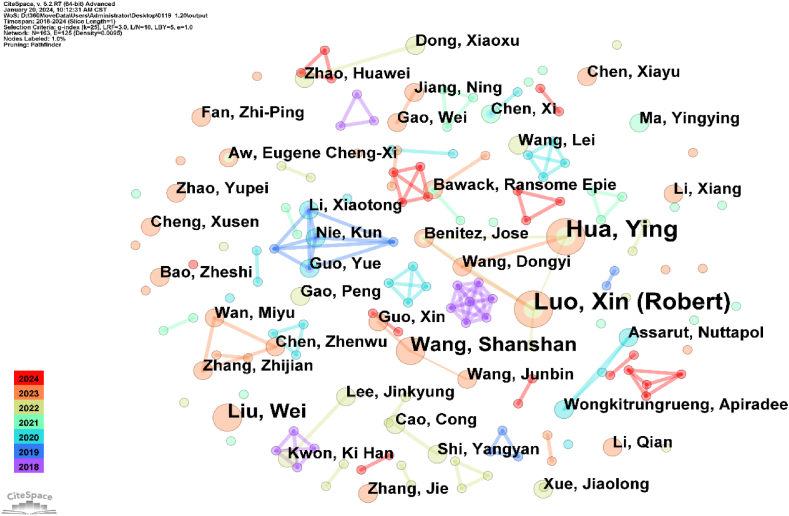
Visualisation of author collaboration network.

The number of papers published in journals partially indicates an author’s academic standing in the field [66]. The author collaboration network depicts the core author group in research and elucidates their collaborative relationships [67]. Fig. 5 illustrates the results; the font and node sizes represent the number of papers published by each author [68]. The connection between nodes represents the cooperative relationship between different authors [69], and the thickness of the connection implies the closeness of cooperation [70]. The most productive and influential authors can be identified by analysing the number of papers published by them in their field of study and the links between authors [71].

As Fig. 5 indicates, the nodes are 163, and the connections are 125, and the overall network density is 0.0095, indicating a robust collaborative network among authors [72]. The vast author collaboration network presented in the figure comprises Luo Xin (Robert), Hua Ying, Wang Dongyi, and others. Based on the number of papers per author, the average number of papers per author is relatively small. The top three authors are Luo Xin (Robert), Hua Ying, and Wang Shanshan. Four authors have published three or more articles. Assessing the collaboration level among authors reveals that the primary authors exhibit a relatively high degree of cooperation. A connection is apparent between highly productive authors and cooperation density, and the primary collaborative network exhibits a relatively high density. However, noteworthily, each author’s centrality is 0.00 (Table 2), indicating a low intensity of collaboration between authors. Therefore, authors are recommended to attend and present at national and international conferences to network with other researchers. Simultaneously, authors are encouraged to join professional associations in their fields, which often provide collaboration opportunities. The sharing of resources and undertaking of collaborative endeavours are promoted, which may contribute to sustainable research in the field of e-commerce.

**Table 2:** Top 10 collaborative authors.

Ranking	Authors	Year	Count	Centrality
1	Luo, Xin (Robert)	2022	4	0.00
2	Hua, Ying	2022	4	0.00
3	Wang, Shanshan	2023	3	0.00
4	Liu, Wei	2023	3	0.00
5	Guo, Yue	2019	2	0.00
6	Nie, Kun	2019	2	0.00
7	Li, Xiaotong	2019	2	0.00
8	Wang, Dongyi	2023	2	0.00
9	Benitez, Jose	2022	2	0.00
10	Assarut, Nuttapol	2020	2	0.00

### 3.3 Co-occurrence analysis

#### 3.3.1 Keyword co-occurrence analysis

**Fig. 6:**
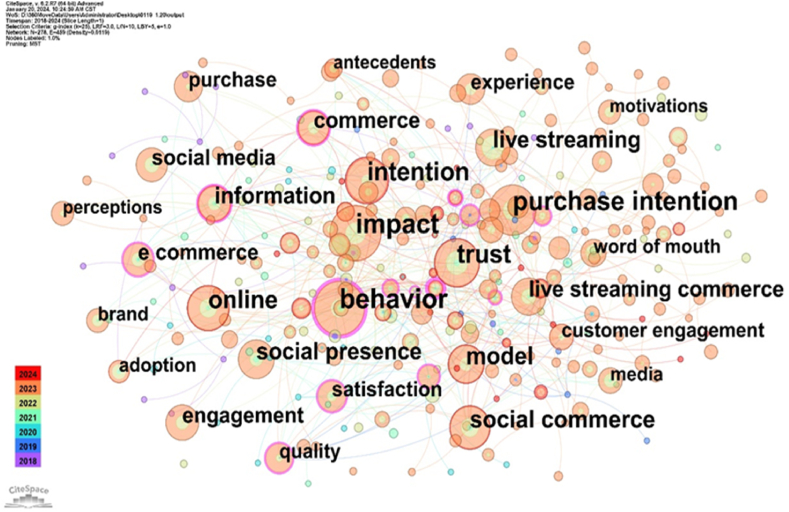
Visualisation of keyword co-occurrence.

Co-occurrence analysis, a primary method, involves extracting bibliographic details, such as keywords and abstracts, from citations [73]. Thereafter, this information is statistically utilised to create an insightful knowledge graph [74]. By examining high-frequency keywords, we can identify research hotspots in a specific period [75]. We extracted keywords based on a set threshold and identified 278 high-frequency keywords, forming 459 connections. Fig. 6 illustrates a keyword co-occurrence map.

As Fig. 6 depicts, the sizes of the nodes and text were proportional to the frequency of keyword occurrences. Interconnections between nodes signify associations established during various periods [71]. The thickness and density of these connections reflect the intensity of keyword co-occurrence [76]. ‘impact’ stands out as the largest node, and ‘behaviour’ and ‘purchase intention’ come second. Judging from the calculated period, keywords such as ‘trust’, ‘model’, ‘live streaming’, ‘information’, and ‘commerce’ appeared earlier. More recently, keywords such as ‘constructs’, ‘deal proneness’, ‘collectivism’, ‘consumer switch’, and ‘curvilinear effect’ have appeared and may become new directions for future research.

The betweenness centrality of keyword occurrence is a crucial indicator for assessing research hotspots in this field. Additionally, it provides a fundamental basis for gauging the focal points of scholarly attention [77]. Evaluating the betweenness centrality index, indicative of the promotional impact of nodes (Table 3), ‘community’, ‘behaviour’, and ‘information’ have strong linkage with other popular keywords, indicating their frequent association with different keywords.

**Table 3:** Top 10 Keyword co-occurrence.

Ranking	Keyword	Count	Centrality	Year
1	community	9	0.35	2018
2	behaviour	81	0.28	2020
3	information	39	0.16	2018
4	consumer	7	0.15	2021
5	attitude	7	0.14	2021
6	commerce	34	0.12	2018
7	ecommerce	32	0.12	2018
8	quality	24	0.12	2019
9	product	11	0.12	2018
10	motivation	3	0.12	2018

In conclusion, keyword co-occurrence provides a clear picture of the evolution in this domain. Future research hotspots could be identified by focusing on these co-occurring keywords.

#### 3.3.2 Keyword cluster analysis

**Fig. 7:**
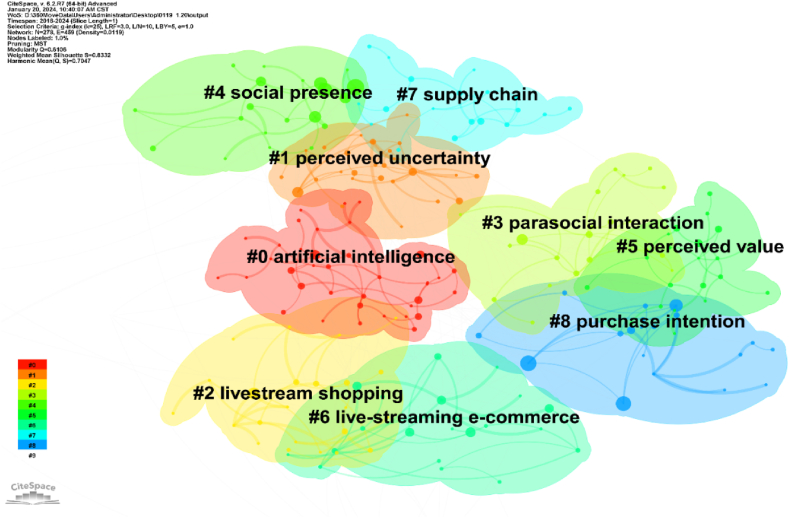
Keywords cluster of LSC research.

Keywords that constitute a vital component of scholarly articles, summarising the study’s core, are frequently used to examine prevalent issues within a specific field [78]. Based on this, this study uses the log-likelihood ratio (LLR) algorithm to cluster keyword co-occurrences to intuitively reflect hot research topics. Fig. 7 presents the keyword clustering view using coloured blocks, which contain clustered keywords representing the clustered area. Nodes N = 278, number of connections E = 459, and network density = 0.0119. The size of the module value Q is related to the node density. The larger the Q value, the better the clustering effect, which can be used for scientific cluster analysis [79]. The size of the average silhouette value S can be used to measure the homogeneity of clustering [80]. The larger the value of S, the higher the homogeneity of the network, which indicate that clustering has high credibility [81].

As Fig. 7 depicts, Q = 0.6106, a pronounced clustering effect within the network structure, S = 0.8332, indicates high homogeneity and satisfactory classification of distinct clusters [82]. These co-occurring keywords cover multiple topics. The figure shows the top 9 clusters, headed by ‘artificial intelligence (#0)’, ‘perceived uncertainty (#1)’, and ‘livestream shopping (#2)’. The average year of the top 5 clusters is around 2020–2022, indicating the maturity of LSC during this period [83]. The largest cluster is ‘artificial intelligence (#0)’ in the year 2020, which contains 37 keywords. (1) Some clusters focus on the process and behaviour of consumer purchase decisions, such as perceived uncertainty (#1), parasocial interaction (#3), social presence (#4), perceived value (#5), and purchase intention (#8). Different consumers, platforms, and live streamers exert different effects on consumers’ purchase intentions in the context of LSC. Therefore, paying attention to the process and behaviour of consumers’ purchase decisions is critical to merchants and platforms. For example, a paper on purchase intention (#8) explored the role of flow experience and social interaction on consumers’ purchase intention from LSC consumers’ perspective [28]. Deng, Lin, and Jiang (2023) [31] explored the mechanism of the role of consumer characteristics on consumer impulse buying behaviour. (2) Some clusters focus on digital commerce and technology integration such as artificial intelligence (#0), livestreaming shopping (#2), livestreaming e-commerce (#6), and supply chain (#7). For instance, a paper considering the novelty of AI live streaming incorporated into cross-border operations deserves to be examined to demonstrate the trade-offs between AI and widely adopted live streaming of key opinion leaders for global brands, developing a cross-border competing model.

Table 4 presents the clustering analysis of keywords in LSC. Keyword cluster analysis proves that the theme of the field is dynamically evolving, covering multiple disciplines such as psychology, information and communication. We recommend that researchers integrate more disciplines into this field in the future.

**Table 4:** Clustering analysis of keywords in LSC.

Cluster ID	Cluster name	Main keywords	Year	Size
#0	artificial intelligence	artificial intelligence; live shopping; cluster analysis; product involvement; voice shopping	2020	37
#1	perceived uncertainty	perceived uncertainty; livestreaming commerce; cognitive assimilation; usefulness; live streaming participation	2021	26
#2	livestream shopping	livestream shopping; consumer engagement; perceived risk; social overload; isc	2020	23
#3	parasocial interaction	parasocial interaction; parasocial relationships; live streaming; live streaming commerce; intention	2022	22
#4	social presence	social presence; streaming media; consumer behaviour; physical presence; perceived value	2020	21

#### 3.3.3 Research evolution path analysis

**Fig. 8:**
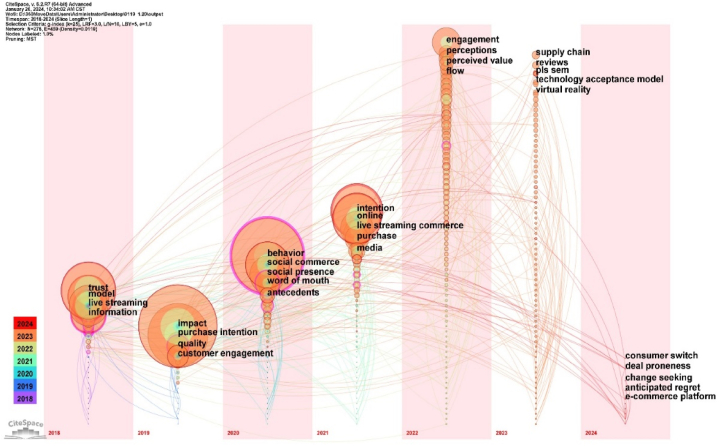
Research time-zone map of LSC.

The time-zone map primarily visualises the evolution of document keywords and their interrelationships over time and space, clearly displaying them in two-dimensional coordinates with time as the horizontal axis [84], as Fig. 8 depicts. In this diagram, the node size indicates the frequency of keyword occurrence; the node year indicates the time when the keyword first appeared, and the connection between nodes indicates that different keywords appear in an article simultaneously, indicating the time between different periods [85]. Transmission relationships and evolutionary processes, combined with the number of papers published over the years, can be used to explore the focus of popular research periods and indicate the period or stage in which the field is situated [86]. Fig. 8 highlights the largest node in the relevant literature, that is, ‘impact’ introduced in 2019. High-frequency keywords in early research include ‘trust’, ‘model’, and “information”. The relevant concepts studied have a long span and broad scope of influence. Relevant research continues, and different conceptual frameworks are gradually being introduced [87]. New conceptual developments have introduced new keywords, including ‘consumer switching’ [88,89] and ‘transactional propensity’ [90,91].

Some detailed findings are presented subsequently. (1) The keyword ‘impact’ became prominent in 2019, despite LSC gaining traction earlier indicating a delayed recognition of the importance of measuring the effects of LSC. Early research focused on broad foundational concepts such as ‘trust’, ‘model’, and ‘information’. However, more recent studies have shifted focus to specific consumer behaviours, such as ‘consumer switching’ and ‘transaction propensity’. This shift suggests a lack of continuity and integration between the foundational aspects and emerging consumer behaviour studies. (2) The shift towards keywords such as ‘consumer switch’, ‘anticipate regret’, ‘change seeking’, and ‘deal proneness’ indicates a broadening of research focus from platform operation to specific behaviours and decision-making processes of consumers engaged in LSC.

#### 3.3.4 Keyword bursts analysis

**Fig. 9:**
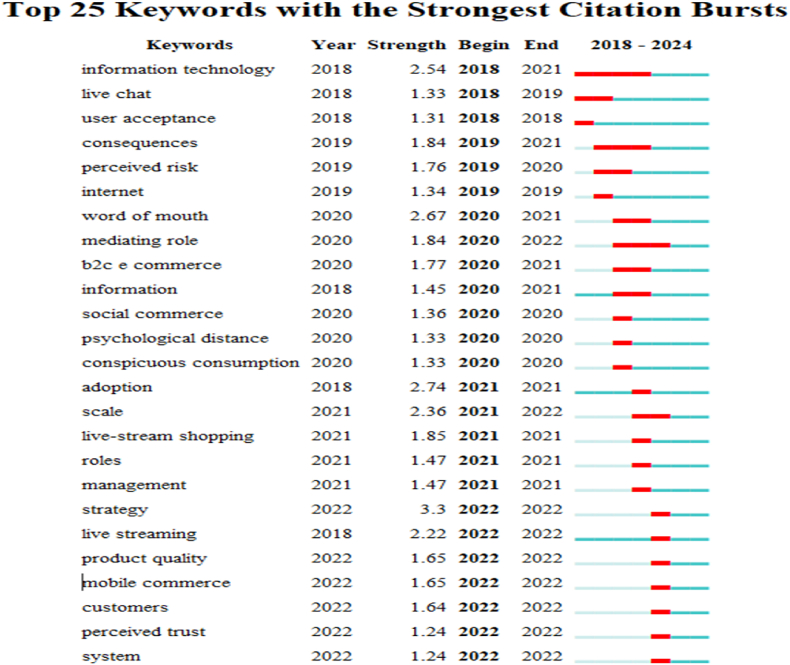
Top 25 keywords with the strongest citation burst.

Fig. 9 shows the words that emerged over the past six years. The first year indicates the beginning of an upward trend in the frequency of the respective keywords, whereas the last year indicates when the frequency stabilises [92]. The intensity of an occurrence measures the degree of sudden surge in frequency during the appearance of a keyword, which is usually a sign of popularity [93]. These characteristics typically occur concurrently. The red bars correspond to the duration of an emerging word [94]. Fig. 9 shows the Top 25 keywords with the strongest citation bursts.

From the time series perspective, ‘information technology’, ‘live chat’, and ‘user acceptance’ were the earliest keywords to emerge. From the emergence duration perspective, ‘information technology’, ‘consequences’, and ‘user acceptance’ were the hotspots of prior research. Additionally, from the emergence duration perspective, ‘information technology’, ‘consequences’, and ‘mediating role’ have longer durations, indicating that they have been the hotspots for a long time. Concerning the strength of the emergent words, ‘strategy’ (Strength = 3.3), ‘adoption’ (Strength = 2.74), and ‘word of mouth’ (Strength = 2.67) have particularly high emergence strengths, indicating that the frequency of their occurrence has changed significantly. Overall, ‘information technology’ and ‘consequences’ show strong emergence and a high frequency of occurrence, making them the most recent emerging research hotspots.

In summary, these time zones and keyword bursts provide insights into the different hotspots at different times. Scholars can continue to conduct more effective research based on the current topics of interest.

## 4 Discussion

### 4.1 Current challenges

Based on the above analysis, LSC is an interdisciplinary research field encompassing various topics that are likely to generate new interdisciplinary issues. Current research faces the following main challenges that deserve readers’ attention: (1) It is more difficult to improve audience engagement and conversion rate. Live content lacks excitement and interactivity, and viewers may quickly lose interest, resulting in a high churn rate in a live stream. It is a major challenge for merchants and live streamers to continuously create high-quality, interesting, and informative words to attract and convert viewers. (2) Consumers’ buying behaviour is harder to understand. Consumers in the LSC environment have different cognitions, attitudes, positions, cultures, experiences, and regions. This diversity challenges the creation of universally applicable theories and marketing strategies. This makes the sustainable development of LSC a challenge. (3) Consumer trust, privacy, and security are compromised. The collection and use of consumer data in LSC has raised concerns about privacy and data security. The increase in fraudulent activities, such as false promotions and counterfeit products, can undermine consumer confidence. A key challenge for platforms and merchants is to ensure compliance with relevant laws and regulations while maintaining consumer trust.

### 4.2 Future research characteristics

As LSC has steadily increased, this surge has led to the emergence of new and crucial research directions, contributing to a more diversified research focus. Therefore, subsequent research should carefully consider the evolving research directions outlined below. (1) The research domain is poised to become more interdisciplinary, incorporating various theories from informatics, communication, and psychology. Interdisciplinary research trajectories integrate diverse field findings, fostering cross-disciplinary communication to comprehensively grasp the essence of LSC. They broaden the research perspectives by delving into its evolution, influence, and future outlook from various disciplinary angles. These trajectories foster innovative crossovers, sparking new methodologies and insights. We recommend that readers study corporate management strategies more comprehensively; explore how digital technologies, such as AI and big data, optimize the LSC experience; and study strategies to alleviate consumers’ concerns about privacy and security in live streaming transactions. (2) Research topics are poised to become more intricate and encompassing. Consumer motivations for engaging in LSC will no longer monopolise this research field; specifically, LSC technological innovation grounded in user experience, supply chain management in LSC, and psychological mechanisms influencing consumer behaviour will emerge as focal points. We recommend readers continue to focus on consumers’ cognition and decision-making behaviour, explore consumers’ purchase decision-making process, and improve user conversion rate and satisfaction. (3) We recommend that readers increase their focus on local supply chain practices. The importance of applying theory to guide effective LSC marketing practices will be reinforced, with an emphasis on customer and live streamer-driven approaches.

### 4.3 Study implications

Theoretically, this study provides important insights for researchers and practitioners. First, we describe the present state and research focal points in LSC and predict future research directions, helping researchers grasp frontier research directions and focus on research hotspots in this field. Practitioners can draw inspiration from our study and gain insights into the cutting-edge trends of digital technology reshaping LSC. Second, this study aids researchers and practitioners to comprehensively understand the current landscape of applying new methods and management models in the field. Finally, this study details the organisations and authors who significantly contributed to LSC research. These results should guide researchers and practitioners in identifying potential areas of collaboration and obtaining valuable information.

Practically, this study helps platforms understand the industry’s development trends and dynamics and guide their future development strategies. Moreover, it can provide valuable references for platforms to help users optimize their services, improve their functions, enhance user experience, and increase user stickiness. Merchants can use the research results to understand the industry’s hotspots and user needs, guiding them to formulate more effective marketing strategies and maintain their competitive advantages. The study helps consumers understand the development status and trends of LSC, choose better-quality platforms and products, and improve their shopping experience and satisfaction.

In summary, this study is relevant for scholars, platforms, merchants, and consumers, as it can provide guidance and inspiration and promote the industry’s healthy development and sustainability.

## 5 Conclusions

In this study, we used CiteSpace to undertake a bibliometric analysis to examine evolutionary trends in LSC, introducing a fresh perspective for evaluating the research progress. Several noteworthy findings were drawn, which are elucidated subsequently. (1) In the past six years, the number of publications has fluctuated, indicating that scholarly interests and focus vary at different stages. (2) Collaboration networks between authors and institutions are relatively close. Globally, the top collaborating institutions are concentrated in China, indicating that the scope of collaboration in LSC research is primarily in China. (3) Concerning co-occurrence and knowledge evolution, ‘influence’, ‘consumer behaviour’, and ‘purchase intention’ are not only well received but also currently hold a dominant position. Additionally, the keywords with the strongest citation bursts showed that the topics are dynamic and evolving, involving multiple disciplines. Moreover, these keywords indicate that the research is related to psychology and informatics.

This study has several innovative aspects. (1) From the knowledge perspective, we provide a comprehensive analysis of the research and development trajectory of LSC, addressing the limitations of previous studies that lacked thoroughness and comprehensive analysis in this research domain. (2) From the theoretical perspective, we establish and elucidate a research framework for LSC, thereby enhancing the knowledge base. Additionally, we present our study’s results using visual figures. This study provides a comprehensive and dynamic understanding of the knowledge framework and its evolution. This simplifies the explanation of the LSC for future scholars and practitioners. (3) From the practical perspective, we identify research gaps and key concerns and underscore the characteristics of future research to enable future scholars and practitioners to grasp specific basics more quickly and gain insights for future research.

However, this study has some limitations. (1) This study relied solely on data from the SSCI and SCIE within the WoS database, without incorporating data from other reputable databases, such as Scopus and EBSCO. This limited the quantity and breadth of documents, thus potentially reducing the statistical results’ accuracy, which might have precipitated bias in the analysis. (2) This study exclusively considered publications in English and overlooked those in other languages. (3) Owing to the data collection cutoff on 18 January 2024, we did not consider the most recent literature-writing period, which might have affected the analytical results.

In future studies, we plan to improve these limitations by increasing the size and scope of the original sample, using data from well-known databases such as Scopus and EBSCO. More recent papers published in other languages should be selected for future research to address these limitations. Finally, we aim to increase the time span by researching the historical context of the field.

## Ethics declarations

Review and approval by an ethics committee were not needed for this study because this study did not involve animal or human experiments.

## Data availability statement

All data associated with this study wasn’t deposited into a publicly available repository, because it will be made available on request from the corresponding author.

## Funding statement

This research received no specific grant from funding agencies in the public, commercial, or not-for-profit sectors.

## CRediT authorship contribution statement

**Yi Huang:** Writing – original draft, Visualization, Software, Conceptualization, Resources, Writing – review & editing. **Nurkhalida Makmor:** Writing – review & editing, Supervision, Project administration. **Siti Hajar Mohamad:** Writing – review & editing, Supervision, Project administration.

## Declaration of competing interest

The authors declare that they have no known competing financial interests or personal relationships that could have appeared to influence the work reported in this paper.
